# The Role of Cognitive Stimulation in the Home and Maternal Responses to Low Grades in Low-Income African American Adolescents’ Academic Achievement

**DOI:** 10.1007/s10964-020-01217-x

**Published:** 2020-04-06

**Authors:** Cecily R. Hardaway, Emma M. Sterrett-Hong, Natacha M. De Genna, Marie D. Cornelius

**Affiliations:** 1 Department of African American Studies, University of Maryland, 1119 Taliaferro Hall, College Park, MD 20742 USA; 2 Kent School of Social Work, University of Louisville, Louisville, KY USA; 3 Department of Psychiatry, University of Pittsburgh School of Medicine, Pittsburgh, PA USA

**Keywords:** Parental involvement, Parenting, Adolescents, Cognitive stimulation, Academic achievement, African American

## Abstract

Parental involvement in education has generally been shown to foster adolescent academic achievement, yet little is known about whether two important forms of parental involvement—how parents respond to academic underachievement and how parents provide cognitive stimulation in the home—are related to academic achievement for African American adolescents. This study uses two waves of data to evaluate whether these forms of parental involvement are related to future academic achievement for low-income African American adolescents and whether there are gender differences in these associations. African American mothers and adolescents (*N* = 226; 48% girls) were interviewed when adolescents were ages 14 and 16. Mothers of girls reported higher mean levels of punitive responses to grades than mothers of boys, but child gender did not moderate associations between parental involvement and academic achievement. Cognitive stimulation in the home was related to changes in academic achievement from 14 to 16 years of age, controlling for age 14 academic achievement. This study provides evidence that nonpunitive responses to inadequate grades and cognitive stimulation at home are linked to academic achievement among African American adolescents.

## Introduction

Parental involvement in education has been defined as “parents’ work with schools and with their children to benefit their children’s educational outcomes and future success” (Hill et al. 2004, p. 1491). The literature on parental involvement primarily focuses on home involvement (e.g., help with homework), school involvement (e.g., volunteering at school), and academic socialization (e.g., communication of expectations for academic success), with the preponderance of studies showing that parental involvement in education is associated with positive academic and psychosocial outcomes for youth (Benner et al. 2016; Hill and Tyson 2009; Jeynes 2016; Wang et al. 2014). Despite the fact that parental involvement may include parental responses to grades (e.g., talking to the child’s teacher, punishing the child, or talking with the child), few empirical studies focused on parental involvement have included this construct.

### Academic Achievement among African American Adolescents from Low-Income Families

In addition, the role of distinct forms of parental involvement in academic achievement among adolescents from low-income backgrounds, including youth whose mothers were teenagers when they were born, is also an important area of inquiry. Studies report that low-income parents generally have lower levels of involvement in education (Wang and Sheikh-Khalil 2014) and that their involvement declines more sharply than parents of higher SES during adolescence (Wang et al. 2014). At the same time, low-income adolescents are more likely than their peers to see steep declines in their grades across adolescence (Wang et al. 2014). More research is needed on maternal involvement in education among teenage mothers because, on average, their children have lower academic achievement than the children of older women (Francesconi 2008; Shaw et al. 2006) and are more likely to drop out of school (Addo et al. 2016). Low maternal involvement in education may be one factor contributing to lower levels of educational attainment in the offspring of teenage mothers. This may be especially true for younger mothers from more disadvantaged backgrounds who lack the education and resources to provide access to higher-quality educational opportunities and advocate for their child’s education (SmithBattle 2007). Given the apparent salutary effects of parental involvement (Hill and Tyson 2009; Jeynes 2007; Pomerantz et al. 2007), it is important to home in on the aspects of parental involvement that are most effective at promoting the academic achievement of low-income youth.

### Parental Responses to Grades

For African American parents, involvement in education takes place in an educational landscape in which African American students, on average, have lower levels of academic achievement than their peers in other racial/ethnic groups (Taylor et al. 2018). School segregation (Reardon 2016), racial disparities in school discipline (Pearman et al. 2019), and low teacher expectations for African American students (McKown and Weinstein 2008), among other factors, help fuel these gaps in achievement. As a consequence of the disadvantaged status of African American students in the American educational system, a disproportionate number of African American parents will be in a position to respond to academic underachievement on the part of their children.

Parental responses to grades, particularly grades that are poor or lower-than-expected, are an important aspect of parental involvement to consider, as how parents respond to grades may support or undermine later academic achievement (Robinson and Harris 2013; Tang and Davis-Kean 2015). This type of parental involvement in education may be qualitatively different and may have different consequences compared to involvement that is not triggered by academic difficulties (Robinson and Harris 2013). Outcomes associated with parental responses to grades are especially salient during adolescence—a time when parents’ oversight of adolescents’ academic affairs may clash with adolescents’ increasing desire for autonomy and self-determination (McElhaney et al. 2009).

Self-determination theory helps explain associations between parental involvement and academic achievement. This theory posits that humans have a fundamental need for autonomy and that meeting this need helps individuals develop autonomous motivation and internalize the value of behaviors necessary for functioning successfully in a variety of domains (Deci and Ryan 2008). When applied to academic achievement, self-determination theory suggests that autonomy-supportive parenting helps promote achievement by fostering autonomous motivation and helping children develop the self-regulatory skills necessary to perform activities related to academic success without prodding (Vasquez et al. 2016). Autonomy-supportive parents use explanations and reasoning, take their child’s perspective, encourage choice, and eschew coercion and control (Joussemet et al. 2008). In contrast, parents that undermine autonomy use coercion and control in ways that erode motivation and self-regulation (Grolnick et al. 2014).

In line with self-determination theory, parental involvement, when implemented in ways that support autonomy, can help foster autonomous motivation, ultimately leading to better academic achievement (Affuso et al. 2017). Nonpunitive responsive to low grades and cognitive stimulation in the home likely support autonomy because they involve providing communication, support, and resources that facilitate increased autonomous motivation and help children internalize the value of academic achievement. In contrast, punitive responses to low grades rely more on coercion and control and are likely to dampen motivation and achievement.

Parents’ responses to their children’s poor grades may increase or decrease the likelihood that children’s academic achievement will improve in the future. For example, some parents may engage in activities that support turnarounds in academic achievement, while other parents may punish their child without providing the proper support for improved achievement. Robinson and Harris (2013) found that parents’ endorsement of punitive responses during primary school (e.g., punish the child, limit child’s non-school activities) were negatively related to achievement during secondary school, and parents’ endorsement of nonpunitive responses during primary school (e.g., contact child’s teacher or principal, spend more time helping child with homework, keep a close eye on child’s activities, tell child to spend more time on schoolwork) were positively related to achievement during secondary school, controlling for prior achievement. Similarly, endorsement of punitive responses but not nonpunitive responses at ages 11–13 were negatively related to adolescent academic achievement five years later in another study (Tang and Davis-Kean 2015). However, the link between punitive responses and academic achievement did not remain once prior academic achievement was controlled. Thus, endorsement of punitive parenting practices in response to grades was related to academic achievement but was not related to changes in academic achievement over a five-year period. The mixed results of these two studies suggest that more research on relations between parental responses to underachievement and future achievement is needed.

Although only a few studies have directly examined punitive responses to grades (Robinson and Harris 2013; Tang and Davis-Kean 2015), the literature on punitive parenting, more broadly, provides evidence that this form of punitive parenting may be negatively related to grades. Previous literature suggests that punitive parenting is associated with socioemotional adjustment problems (e.g., defiance and aggression; Roche et al. 2007, 2011) and socioemotional adjustment problems are associated with poor academic achievement (Okano et al. 2020). Therefore, it is plausible that socioemotional adjustment problems may be one mechanism linking punitive parenting to academic outcomes. However, the literature on punitive parenting largely focuses on physical discipline (e.g., Roche et al. 2011) and does not include a broader array of parenting behaviors that could also be characterized as punitive (e.g., taking away privileges, grounding, removing toys/gadgets). This literature also does not specifically address how parents respond when children’s academic achievement is less than expected. As a result, whether punitive responses to grades are associated with negative outcomes in ways that parallel punitive parenting more broadly is not known.

Questions also remain regarding how punitive parenting may differentially affect subpopulations. There is some evidence that the effects of punitive parenting may vary, depending on factors such as race and neighborhood context (Lansford 2010; Roche et al. 2007). African American parents typically use firmer discipline strategies and exercise greater levels of behavioral control (e.g., more restrictive rules and greater levels of monitoring and supervision) than other parents (McLoyd et al. 2019), and some studies have shown that punitive parenting is less strongly related to negative outcomes for African American youth compared to White youth (Lansford 2010). However, as stated earlier, the bulk of this literature has focused on physical discipline, so whether these findings apply to other forms of punitive parenting is unclear. It is also unclear whether African American parents respond similarly to inadequate grades and child misbehavior.

However, studies of punitive and nonpunitive responses to grades have not been undertaken with solely African American samples. Research suggests that parental expectations for high academic performance are a predictor of academic achievement among African American adolescents (see Jeynes 2016, for a meta-analysis), and African American parents tend to have high educational expectations for their children (Hayes 2011; Spera 2006; Suizzo and Stapleton 2007). The variety of verbal and behavioral responses they have when their expectations are not met and how those responses are associated with future academic achievement is an important gap in the literature. Racial comparative studies suggest that African American parents and those with lower levels of education are more likely to respond punitively to lower-than-expected grades than are other parents (Robinson and Harris 2013; Tang and Davis-Kean 2015), and past studies have shown that endorsement of punitive responses to poor grades is associated with lower levels of achievement (Robinson and Harris 2013; Tang and Davis-Kean 2015). Punitive responses to low grades have also been linked to the gap in test scores between African American and white students (Robinson and Harris 2013). Given that low-income African American adolescents are at particularly high risk for low academic achievement, within-group studies that hone in on modifiable predictors are needed (Gutman et al. 2017). Understanding the impact of parental responses to lower grades on subsequent academic achievement will result in a more comprehensive understanding of parental influence on academic performance and additional targets for intervention among African American families.

### Cognitive Stimulation at Home

Cognitive stimulation in the home is another form of parental involvement in education that overlaps with existing definitions of home-based parental involvement in education (Hill and Tyson 2009). Cognitive stimulation in the home includes having educational materials and engaging in enrichment activities thought to promote cognitive development and learning. Cognitive stimulation in the home declines as children grow older (Simpkins et al. 2009), and much of the literature on this topic has focused on very young children. These studies have found that cognitive stimulation in the home predicts vocabulary (Chapin and Altenhofen 2010), reading ability (Tucker-Drob and Harden 2012), cognitive skills (Jeon et al. 2014), mathematics skills (Powell et al. 2012), and executive function (Baker and Brooks-Gunn 2019). Although research suggests that cognitive stimulation in the home has a positive influence on academic achievement across adolescence (Eamon 2005; Simpkins et al. 2009; Tang and Davis-Kean 2015), studies elucidating specific pathways are lacking. It is likely that cognitive stimulation in the home functions similarly during childhood and adolescence by fostering academic achievement both directly and indirectly through helping adolescents develop important skills and abilities that are necessary to perform well in school.

Despite evidence of links between cognitive stimulation in the home and adolescent academic achievement, very little research has focused on cognitive stimulation in the home as a predictor of academic achievement specifically for African American adolescents. In a cross-sectional study of African American children (10 to 14 years), cognitive stimulation in the home was positively related to achievement test scores (Mandara et al. 2010). In a longitudinal study of children from a wider age range (0 to 13 years), cognitive stimulation in the home was related to academic test scores for African American children and the strength of this relationship was consistent across age groups (Bradley et al. 2001). Given the limited number of studies related to parental cognitive stimulation conducted with African American adolescents and the importance of academic performance in high school for future career success (Spengler et al. 2018), parental provision of cognitive stimulation to adolescents in high school deserves empirical attention.

### Gender Differences in Parental Involvement in Education

A dearth of studies has focused on gender differences with regard to parental involvement in the education of African American adolescent sons and daughters. There is evidence from a study of 10 to 14 year old African American children that girls experience more cognitive stimulation than boys (Mandara et al. 2010). Previous studies that focused on punitive and nonpunitive responses to grades have examined racial differences in parental responses but have not examined how mothers respond to inadequate grades on the part of girls compared to boys (Robinson and Harris 2013; Tang and Davis-Kean 2015). Some research suggests that African American mothers use different parenting strategies with boys and girls. Different patterns in parenting by child gender are not unique to African Americans (Shanahan et al. 2007); however, a small group of noteworthy studies have specifically focused on African American mothers and their children. These studies have shown that African American mothers are more emotionally responsive to girls, monitor girls more, enforce more rules directed at girls’ behavior, and have higher educational and career expectations for girls (Mandara et al. 2012; Varner and Mandara 2013a, 2013b). On the other hand, mothers report more conflict with boys than girls (Varner and Mandara 2013a).

Concerns about racial discrimination, specifically that racial discrimination will have a more negative impact on the future well-being of boys as opposed to girls, have been identified as one explanation for variation in parenting practices by child gender (Varner and Mandara 2013a). Although studies indicate that African American mothers parent boys and girls differently, it is unclear whether or how these differences in parenting are related to disparate outcomes for boys and girls. Some work does suggest that differential socialization and parenting for African American boys and girls may, in part, account for some of the gender disparity in achievement (Varner and Mandara 2013b). More work is needed to understand whether different forms of parental involvement are differentially related to academic achievement over time for African American boys and girls. Addressing this gap in the literature is particularly important given the well-documented disparities in academic achievement between boys and girls (Duke 2017).

## Current Study

The current study is a longitudinal, within-group analysis of links between parental involvement and low-income African American adolescents’ academic achievement. It investigates three aspects of parental involvement, including the extent to which parents respond nonpunitively and punitively to lower-than-expected academic achievement and cognitive stimulation in the home. One aim of this study was to examine whether parental involvement in education is related to academic achievement for low-income African American adolescents. It was hypothesized that nonpunitive responses to inadequate grades would be associated with better academic achievement and that punitive responses would be associated with poorer academic achievement over time. The broader parental involvement literature suggests that nonpunitive behaviors are beneficial for academic achievement (Hill and Tyson 2009; Jeynes 2016). In contrast, the literature on punitive parenting suggests that punitive responses to lower grades will either be ineffective at promoting academic achievement or detrimental to academic achievement. Punitive parenting and punitive disciplinary strategies, more broadly, are associated with negative socioemotional adjustment outcomes for children and youth (Robinson and Harris 2013; Roche et al. 2007, 2011; Tang and Davis-Kean 2015). Although little attention has been paid to cognitive stimulation in the home and its relation to academic achievement for African American adolescents, the broader literature points to salutary effects for younger children (Ansari and Gershoff 2016; Powell et al. 2012), and a small number of studies have also suggested benefits for adolescents (Longo et al. 2017; Mandara et al. 2010; Tang and Davis-Kean 2015). Therefore, cognitive stimulation in the home is expected to be positively associated with academic achievement for African American adolescents.

The second aim of the study was to examine gender differences in levels of parental involvement and differences in the relationships between each form of parental involvement and academic achievement. Thus far, few studies have examined gender differences in parental involvement and its association with academic achievement. Specifically, research focused on how parents respond to poor grades has not examined gender differences. However, one study that focused on a sample of African American adolescents, ages 10 to 14 years old, found evidence of gender differences in levels of cognitive stimulation in the home, with girls experiencing more cognitive stimulation than boys (Mandara et al. 2010). Further, a small number of studies have reported differences in African American mothers’ parenting practices and socialization of boys compared to girls (Mandara et al. 2012; Varner and Mandara 2013a, 2013b). Although the literature suggests that gender differences in parental involvement and its relationship to academic achievement are plausible, the literature base is not sufficient to offer specific hypotheses regarding gender differences in the levels of each type of parental involvement or how different types of parental involvement may be differentially related to academic achievement by gender. Therefore, analyses focused on gender differences were treated as exploratory.

## Methods

### Study Design

This study draws on data from the Teen Mother Study, part of a consortium of studies focused on the long-term effects of prenatal substance use on children’s development. This study was a naturalistic examination of substance use during pregnancy among teenagers and the effects of substance exposure on offspring outcomes. Pregnant adolescents (12–18 years old; 69% African American, 31% white) were recruited from a prenatal clinic from 1990 to 1994. All pregnant adolescents who visited the prenatal clinic were eligible for inclusion in the study, and substance use was not used as a selection criterion. Adolescents participated in both a prenatal visit during the first half of pregnancy and another visit at delivery. The participation rate at the start of the study was 99%. At the delivery phase, there were 413 live-born singletons. Mothers and their children participated in follow-up visits when children were ages 6 (1995–2000), 10 (2000–2005), and 14 and 16 (2005–2011). Data from African American mothers at ages 14 to 16 were examined as academic performance at these stages is most related to future career success and financial stability (Spengler et al. 2018). Each phase of this study was approved by the Institutional Review Boards of the University of Pittsburgh and the Magee Womens Hospital, the location of the prenatal clinic, approved the prenatal and delivery phases.

Mothers and children were assessed by trained interviewers and completed self-report measures in offices at the University of Pittsburgh. At each phase of the study, mothers reported on their sociodemographic information, parenting, the child’s behavior, and the home environment. At the age 14- and 16-year assessments, the children reported their own behavior. Reports on maternal substance use and the growth and behavioral outcomes of the children have been provided elsewhere (e.g., Cornelius et al. 2002, 2012; De Genna et al. 2009).

The present study focuses on the African American mothers and their offspring at the 14- and 16-year follow-up phases. Overall sample sizes at 14 and 16 years were 318 and 334, representing retention rates of 77 and 81% of the total number at births, respectively. The final sample for the current study is comprised of 226 African American mother–child pairs. White mother–child pairs were not included in this analysis, given the aims of the study. Mean maternal age was 33 years (*SD* = 7) at the age 14 follow-up and 35.10 years (*SD* = 7) at the age 16 follow-up. Seventy-nine percent of mothers were unmarried, and 35% were living with a partner at both the age 14- and 16-year follow-ups. Ninety-three percent of mothers had at least 12 years of education. At the age 14 follow-up, the mean monthly household income was $2039 (*SD* = $1491). The offspring sample included 109 girls and 117 boys. Their mean age was 14.44 years (*SD* = 0.58: range = 13.85–16.28) and 16.44 years (*SD* = 0.50: range = 15.93–18.31) at the age 14- and 16-year follow-ups, respectively. Families who were included in the sample for the present study did not significantly differ from those who were not included with respect to annual household income, mothers’ education level, child gender, and employment status at the birth assessment. Participants who were married at the birth assessment were less likely to be included in the analytic sample than those who were unmarried χ^2^ (1) = 7.36, *p* < 0.01, and those included in the analytic sample (*M* = 16.19, *SD* = 1.23) were slightly younger at the child’s birth than those not included (*M* = 16.45, *SD* = 1.14).

### Measures

#### Punitive and nonpunitive responses

At the age 14 follow-up, mothers completed the Home Observation for Measurement of the Environment (HOME; Bradley and Caldwell 1984). They were asked how they would respond if their child brought home a report card with grades that were lower-than-expected. Parents indicated how likely they were to take each of 7 actions on a 5-point Likert scale, ranging from 1 (*not at all likely*) to 5 (*very likely*). Punitive responses (3 items; α = 0.68) included lecture the child, punish the child, and limit or reduce the child’s non-school activities (play, sports, clubs, etc.). Nonpunitive responses (4 items; α = 0.72) included contact the teacher or principal, talk with the child, keep a closer eye on child’s activities, and spend more time helping the child with schoolwork. The mean of the items for punitive and nonpunitive responses were used as the scores.

#### Cognitive stimulation

At the14-year follow-up assessment, mothers completed a 9-item subscale of the HOME that focused on cognitive stimulation in the home (Bradley and Caldwell 1984). Mothers indicated, for example, the number of books in the home, whether musical instruments were present in the home, and whether a family member had taken the child to a museum. Each item was coded 0 (no) or 1 (yes), and the sum of the items was used to compute the final score.

#### Academic achievement

Three variables, assessed at ages 14 and 16 by maternal and adolescent report, were used as indicators of the latent construct academic achievement: subject grades, overall school performance, and school grades. Four items were used to assess subject grades. Mothers indicated on a 4-point scale (0 = *failing*; 1 = *below average*; 2 = *average*; 3 = *above average*) how well their child was doing in each of four subject areas: reading, English, or language arts; history or social science; math; and science. Items were averaged to compute the score for subject grades. One item was used to assess overall school performance. Mothers reported on a 4-point scale how well their child was doing in school overall (1 = *poor*; 2 = *fair*; 3 = *well*; 4 = *very well*). One item was used to assess school grades. Adolescents indicated what grades they earned in school (1 = *mostly Fs*, 2 = *mostly Ds*, 3 = *mostly Cs*, 4 = *mostly Bs*, 5 = *mostly As*).

### Data Analysis and Model Specification

A path analysis was conducted using Mplus version 8.2 to examine the hypothesized relations between the predictors and outcome variable. Model paths were specified a priori based on the existing literature, and alternative structural models were not tested^1^. Maximum likelihood estimation was used to handle missing data (Muthén and Muthén 1998–2017; Schafer and Graham 2002). Three path models were tested. Model 1 tested the hypothesized model, including only a control for maternal education. Model 2 tested the same model as Model 1 with an additional control for age 14 academic achievement. Model 3 tested for gender differences in Model 2. This model was exploratory, given the lack of literature on gender differences in parental involvement in education and its relationship to academic achievement. Models were evaluated based on model fit and the size and significance of the coefficients. Model fit was assessed using several indicators, with nonsignificant chi-square values, RMSEA values of < 0.08, CFI values of ≥ 0.95, and SRMR values of equal to or < 0.08 used as evidence of acceptable model fit (Mueller and Hancock 2019).

Multiple group analysis was used to simultaneously fit the hypothesized path model for girls and boys. These analyses were conducted to examine gender differences in levels of parental involvement (i.e., parental responses to inadequate academic achievement and cognitive stimulation in the home) and whether cognitive stimulation in the home and nonpunitive and punitive responses to lower-than-expected grades were related to academic achievement differentially for boys and girls. Each multiple group model included tests for significant differences in intercepts and paths by child gender.

In analyses not shown, factorial invariance was established for both age 14 and age 16 academic achievement, meaning there were no significant differences in loadings for academic achievement across groups at either time point. Therefore, corresponding loadings for academic achievement were constrained to be equal for boys and girls at age 14 and 16 in models including multiple group analysis. The loading for subject grades was set to one at ages 14 and 16 in order to scale the latent variable. Error covariances for corresponding indicators of academic achievement were allowed to covary across the two time points.

Maternal education 14 years postpartum (number of years of education) was included as a direct path to each age 14 parental involvement variable (punitive responses, nonpunitive responses, and cognitive stimulation in the home) and to age 16 academic achievement. Multiple group models used child gender (operationalized for the study as sex assigned at birth: 0 = *female*, 1 = *male*). Models that included prior academic achievement included a direct path from age 14 academic achievement to age 16 academic achievement. Correlational paths among all age 14 parental involvement variables were also included in all models, and models that included age 14 academic achievement included correlational paths between age 14 academic achievement and each form of parental involvement.

## Results

Descriptive statistics and bivariate correlations are shown in Table 1. Nonpunitive responses and cognitive stimulation in the home were both positively correlated with all three indicators of academic achievement. Punitive responses were not correlated with any of the indicators of academic achievement.

**Table 1:** Descriptive statistics and correlations between study variables

Study variable	1	2	3	4	5	6	7	8	9	10	11	*M*	*SD*
1. Child gender (male)	–												
2. Maternal education	0.13	–										12.85	1.41
3. Subject grades (14)	−0.16*	0.11	–									2.22	0.59
4. Overall performance (14)	−0.16*	0.09	0.67**	–								2.95	0.95
5. School grades (14)	−0.03	0.12	0.52**	0.48**	–							3.80	0.87
6. Nonpunitive responses (14)	−0.05	0.14*	0.32**	0.26**	0.18**	–						4.66	0.52
7. Punitive responses (14)	−0.15*	0.05	0.09	0.01	−0.11	0.45**	–					4.29	0.78
8. Cognitive stimulation (14)	−0.11	0.11	0.17*	0.15*	0.22**	0.29**	0.04	–				4.44	1.84
9. Subject grades (16)	−0.21**	0.15*	0.41**	0.34**	0.30**	0.17*	−0.01	0.23**	–			2.07	0.74
10. Overall performance (16)	−0.20**	0.06	0.40**	0.37**	0.30**	0.16*	−0.02	0.22**	0.72**	–		2.74	1.04
11. Grades (16)	−0.08	0.04	0.35**	0.29**	0.46**	0.05*	−0.08	0.15*	0.51**	0.52**	–	3.65	0.87

Numbers in parentheses represent adolescent age. Correlations between factor indicators are in bold

*p < 0.05; **p < 0.01

**Fig. 1:**
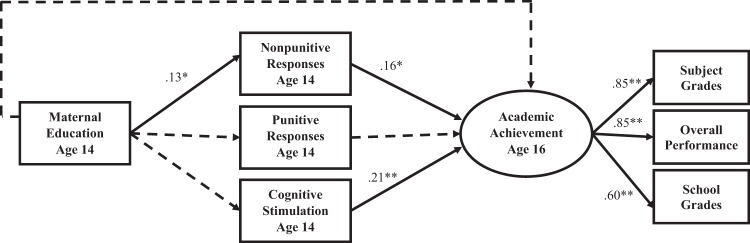
Path model of associations between parental involvement and adolescent academic achievement at age 16 (Model 1). Standardized estimates are presented. Dotted lines indicate paths that are not significant at *p* ≤ 0.05. Correlations between age 14 variables are not shown (see Table 2). “Maternal Education Age 14” denotes mothers’ education at 14 years postpartum. *p < 0.05; **p < 0.01

Model 1 was tested on the full sample of African American adolescents (Table 2 and Fig. 1). This model included maternal education at the age 14 follow-up as a covariate but did not include academic achievement at age 14 as a covariate. Fit indices suggested that the hypothesized model provided good fit to the data (Table 2). Nonpunitive and punitive responses were correlated (*β* = 0.45, *p* < 0.01), and nonpunitive (*β* = 0.28, *p* < 0.01) but not punitive responses were correlated with cognitive stimulation in the home. Nonpunitive responses and cognitive stimulation in the home were positively related to age 16 academic achievement (*β* = 0.16, *p* < 0.05; *β* = 0.21, *p* < 0.01). Punitive responses were not related to age 16 academic achievement, nor was mothers’ educational attainment.

**Table 2:** Parameter estimates and fit indices for models 1 and 2

	Model 1				Model 2			
	*b*	*SE*	95% CI	*β*	*b*	*SE*	95% CI	*β*
Parental involvement to academic achievement								
Nonpunitive responses → AC2	0.20*	0.10	(0.00, 0.39)	0.16	−0.04	0.09	(−0.23, 0.14)	−0.04
Punitive responses → AC2	−0.01	0.06	(−0.22, 0.03)	−0.12	−0.04	0.06	(−0.15, 0.07)	−0.05
Cognitive stimulation → AC2	0.07**	0.03	(0.02, 0.12)	0.21	0.05*	0.02	(0.00, 0.09)	0.14
Controls to academic achievement								
Maternal education → AC2	0.04	0.03	(−0.03, 0.10)	0.08	0.02	0.03	(−0.04, 0.07)	0.04
AC1 → AC2					0.64**	0.09	(0.46, 0.82)	0.54
Maternal education → AC1					0.05	0.03	(−0.00, 0.10)	0.14
Controls to parental involvement								
Maternal education → nonpunitive responses	0.05*	0.02	(0.00, 0.01)	0.13	0.05*	0.02	(0.00, 0.10)	0.14
Maternal education → punitive responses	0.03	0.04	(−0.04, 0.10)	0.05	0.03	0.04	(−0.04, 0.10)	0.05
Maternal education → cognitive stimulation	0.15	0.09	(−0.02, 0.32)	0.11	0.15	0.09	(−0.02, 0.32)	0.11
Correlations								
Nonpunitive responses & punitive responses	0.18**	0.03	(0.12, 0.24)	0.45	0.18**	0.03	(0.12, 0.24)	0.45
Nonpunitive responses & AC1					0.09*	0.02	(0.05, 0.13)	0.34
Nonpunitive responses & cognitive stimulation	0.27**	0.07	(0.14, 0.40)	0.28	0.27**	0.07	(0.14, 0.40)	0.29
Punitive responses & AC1					0.02	0.03	(−0.04, 0.08)	0.05
Punitive responses & cognitive stimulation	0.06	0.10	(−0.13, 0.24)	0.04	0.06	0.10	(−0.13, 0.24)	0.04
Cognitive stimulation & AC1					0.18**	0.07	(0.05, 0.32)	0.20
	χ^2^	*df*	RMSEA	CFI	SRMR			
Model summary								
Model 1	5.12	8	0	1.00	0.02			
Model 2	22.97	23	0	1.00	0.04			

*AC1* academic achievement at age 14 and *AC2* academic achievement at age 16

*RMSEA* Root Mean Square Error of Approximation, *CFI* Comparative Fit Index, *SRMR* Standardized Root Mean Square Residual

*p < 0.05; **p < 0.01

**Fig. 2:**
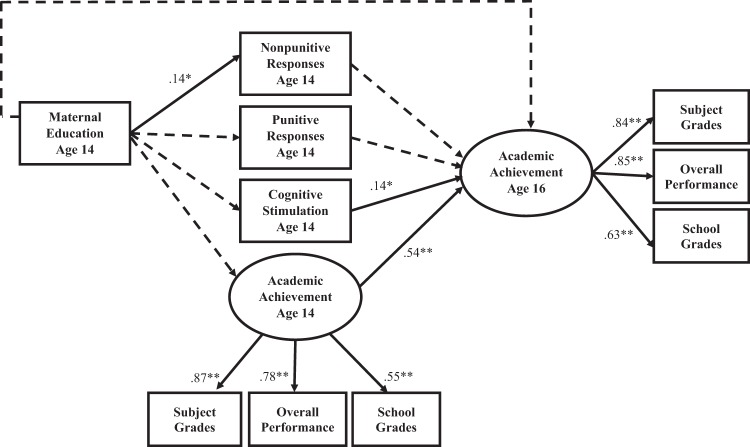
Path model of associations between parental involvement and age 16 academic achievement with control for age 14 academic achievement (Model 2). Standardized estimates are presented. Dotted lines indicate paths that are not significant at *p* ≤ 0.05. Correlations between age 14 variables are not shown (see Table 2). *p < 0.05; **p < 0.01

Model 2 added age 14 academic achievement as a covariate to Model 1 (Table 2 and Fig. 2) to control for the effects of earlier achievement on later achievement. Compared to Model 1, Model 2 was more conservative and allowed for the examination of whether parental involvement was related to changes in academic achievement from ages 14 to 16. Fit indices suggested that the hypothesized model provided good fit to the data (Table 2). In this model, age 14 academic achievement was moderately related to age 16 academic achievement (*β* = 0.54, *p* < 0.01). Controlling for age 14 academic achievement reduced the size of some coefficients and caused some to become nonsignificant. However, age 14 cognitive stimulation in the home remained positively related to age 16 academic achievement (*β* = 0.14, *p* < 0.05). Nonpunitive responses were no longer associated with age 16 academic achievement, and, as in Model 1, punitive responses were not related to age 16 academic achievement.

Model 3 examined gender differences in the hypothesized paths tested in Model 2 (Table 3)^2^. Fit indices suggested that the hypothesized model provided good fit to the data. A test comparing the difference in intercepts of punitive responses by gender revealed that mothers of girls reported higher mean levels of punitive responses to lower-than-expected grades than did mothers of boys (*b* = 2.09, *p* < 0.05). There were no significant gender differences in nonpunitive responses or cognitive stimulation in the home. There also were no significant gender differences in the relationships among any of the three types of parental involvement and age 16 academic achievement. Although cognitive stimulation in the home was related to academic achievement in Model 2, the multiple group analysis (Model 3) showed that there were no gender differences in the strength of the association between cognitive stimulation in the home and academic achievement, meaning cognitive stimulation in the home was similarly related to academic achievement for boys and girls.

**Table 3:** Parameter estimates and fit indices by child gender

	Model girls				Model boys			
	*b*	*SE*	95% CI	*β*	*b*	*SE*	95% CI	*β*
Parental involvement to academic achievement								
Nonpunitive responses → AC2	−0.12	0.14	(−0.39, 0.15)	−0.10	0.05	0.13	(−0.20, 0.29)	0.04
Punitive responses → AC2	−0.07	0.08	(−0.23, 0.10)	−0.08	−0.05	0.08	(−0.20, 0.10)	−0.07
Cognitive stimulation → AC2	0.07*	0.03	(0.01, 0.13)	0.22	0.02	0.02	(−0.04, 0.08)	0.06
Controls to academic achievement								
Maternal education → AC1	0.08	0.04	(−0.00, 0.16)	0.20	0.04	0.03	(−0.03, 0.11)	0.12
Maternal education → AC2	0.04	0.04	(−0.05, 0.12)	0.08	0.01	0.04	(−0.06, 0.09)	0.03
AC1 → AC2	0.73**	0.12	(0.49, 0.97)	0.64	0.54**	0.14	(0.27, 0.82)	0.45
Controls to parental involvement								
Maternal education → nonpunitive responses	0.07	0.04	(−0.00, 0.15)	0.18	0.04	0.03	(−0.02, 0.11)	0.12
Maternal education → punitive responses	−0.05	0.06	(−0.16, 0.06)	−0.09	0.09	0.05	(−0.00, 0.19)	0.17
Maternal education → cognitive stimulation	0.37**	0.13	(0.11, 0.63)	0.26	0.05	0.11	(−0.17, 0.27)	0.04
Correlations								
Nonpunitive responses & punitive responses	0.18**	0.04	(0.11, 0.25)	0.54	0.18**	0.05	(0.09, 0.27)	0.39
Nonpunitive responses & AC1	0.08**	0.03	(0.03, 0.13)	0.36	0.09**	0.03	(0.03, 0.15)	0.32
Nonpunitive responses & cognitive stimulation	0.28**	0.08	(0.12, 0.43)	0.34	0.24*	0.10	(0.04, 0.44)	0.23
Punitive responses & AC1	0.05	0.04	(−0.02, 0.12)	0.16	−0.03	0.04	(−0.11, 0.05)	−0.07
Punitive responses & cognitive stimulation	0.19	0.11	(−0.04, 0.41)	0.16	−0.09	0.14	(−0.38, 0.19)	−0.06
Cognitive stimulation & AC1	0.16	0.09	(−0.01, 0.33)	0.19	0.17	0.10	(−0.03, 0.36)	0.17
	χ^2^	*df*	RMSEA	CFI	SRMR			
Model summary	58.64	52	0.03	0.99	0.06			

*AC1* academic achievement at age 14 and *AC2* academic achievement at age 16

*RMSEA* Root Mean Square Error of Approximation, *CFI* Comparative Fit Index, *SRMR* Standardized Root Mean Square Residual

*p < 0.05; **p < 0.01

## Discussion

Parental involvement in education has been linked to higher levels of academic performance for children and adolescents (Banerjee et al. 2011; Harris et al. 2014; Suizzo et al. 2016). The current study extends the existing literature on parental involvement in education by examining parental responses to grades and cognitive stimulation in the home and focusing on a sample of low-income African American mothers and their adolescent children. Nonpunitive responses to inadequate academic achievement and cognitive stimulation in the home were positively related to later academic achievement, but nonpunitive responses to inadequate academic achievement were not related to later academic achievement when prior levels of academic achievement (age 14) were controlled. Further, there was a longitudinal relationship between age 14 cognitive stimulation in the home and age 16 academic achievement, even when prior academic achievement (age 14) was controlled.

Results showing that cognitive stimulation in the home was related to academic achievement but that nonpunitive responses to grades were not are consistent with prior research by Tang and Davis-Kean (2015). However, unlike Tang and Davis-Kean (2015), which included a diverse sample of primarily white and African American adolescents, the current study found that cognitive stimulation in the home was related to changes in academic achievement over time and that punitive responses to inadequate academic achievement were not related to academic achievement. Past research indicates that African American parents place greater emphasis on behavioral control, tend to be more firm in their disciplinary practices, and endorse more authoritarian beliefs than other groups (McLoyd et al. 2019). Indeed, African American parents are more likely to endorse punitive responses to grades than white parents (Robinson and Harris 2013; Tang and Davis-Kean 2015). Punitive responses, as operationalized in the current study, may not have been sufficiently harsh to negatively impact academic achievement (i.e., lecturing, punishing, and limiting/reducing non-school activities) or these responses may be normative and in line with African American adolescents’ expectations, given that firm parenting styles are more common among African American families, and therefore not detrimental to academic achievement. Further research is needed to understand links between harsh or punitive parenting and academic achievement. Although harsh parenting has been linked to a host of youth mental and physical health conditions (Brody et al. 2003; Cho and Kogan 2016; Kazak et al. 2014), it has been studied to a lesser extent in the context of involvement in education and in relation to adolescent academic achievement among African American families (see Caughy et al. 2017 for an exception).

Whether mothers indicated that they would respond punitively or nonpunitively to low grades, it is important to point out that mothers overwhelmingly indicated a willingness to respond. In analyses not shown, punitive and nonpunitive parental responses were recomputed such that only responses that were “very likely” were counted. Almost 80% of mothers endorsed at least one punitive response, and 95% of mothers endorsed at least one nonpunitive response. Looking at punitive and nonpunitive responses combined, 96% of mothers endorsed one or more responses. Few parents indicated that they were not “very likely” to respond in at least one of the punitive or nonpunitive ways assessed. These statistics point to high levels of involvement on the part of mothers and highlight their desire to address underachievement, whether that is through harsher parenting strategies or approaches that are more supportive.

This study provides compelling evidence for the importance of cognitive stimulation in the home for African American adolescents’ academic achievement. It corroborates other studies that have found links between cognitive stimulation in the home and adolescent academic achievement (Eamon 2005; Tang and Davis-Kean 2015), and it extends this literature by focusing exclusively on African American adolescents. Of the forms of parental involvement in education that were examined in the current study, cognitive stimulation in the home was most strongly related to academic achievement over time. These results point to the importance of providing access to enriching materials (e.g., books and musical instruments) and experiences (e.g., visiting museums) for adolescents. Recent research found that cognitive stimulation in the home was the strongest predictor of low-income children’s academic achievement at 54 months, 5th grade, and age 15, after accounting for other parenting variables (i.e., safety and sustenance, socioemotional support, structure, and surveillance; Longo et al. 2017). The current study and previous research point to the importance of not limiting the study of cognitive stimulation in the home to early childhood (Eamon 2005; Tang and Davis-Kean 2015).

Despite the apparent benefits of cognitive stimulation in the home, low-income children typically receive less than their higher-income counterparts (Dearing et al. 2009). Low-income parents face barriers to cultivating a stimulating home learning environment, as some forms of cognitive stimulation require time, resources, and access to transportation that not all parents have (Dearing and Tang 2014). For example, the cost of musical instruments, lessons (e.g., music, dance), museum entrance fees, and tickets to cultural events is prohibitive for some families. Low-income parents may rely more on public goods (e.g., libraries) and free or low-cost activities than more economically advantaged parents, and these institutional resources are often lacking in low-income neighborhoods (Royce 2015).

Further, for African American parents and adolescents, the cultural relevance of cognitively stimulating activities may be particularly important. Activities that combine racial socialization and cognitive stimulation may be even more beneficial for fostering academic achievement, as past research has shown that racial socialization is positively related to African American adolescents’ academic achievement (Brown et al. 2009). Reading books, visiting museums, and engaging in cultural activities that highlight African American art, history, and culture may serve the dual purpose of cognitive stimulation and racial socialization (Caughy et al. 2002). More research on culturally relevant and age-appropriate forms of cognitive stimulation for African American adolescents is needed.

The current study also explored gender differences in levels of parental involvement as well as gender differences in relationships between parental involvement and academic achievement. Although there were no mean differences between boys and girls in cognitive stimulation in the home or nonpunitive responses, mothers of girls reported higher mean levels of punitive responses to lower-than-expected grades than did mothers of boys. This finding is consistent with prior research showing that African American mothers are stricter with daughters and have higher expectations for daughters compared to sons (Varner and Mandara 2013a, b). There were no gender differences in the relations between any of the three types of parental involvement (i.e., parental responses to inadequate academic achievement and cognitive stimulation in the home) and age 16 academic achievement, meaning child gender did not moderate associations between parental involvement and academic achievement. More research is needed to understand whether differences in punitive responses to adolescent boys’ and girls’ grades may impact other areas of functioning and behavior outside of academic achievement (e.g., internalizing or externalizing symptoms). Past research has shown that punitive discipline is related to different outcomes for boys and girls (Roche et al. 2011).

This study points to concrete ways that parents can respond more effectively to their child’s achievement at school and create a home environment that fosters academic achievement. The results of this study suggest that nonpunitive responses to inadequate grades and cognitive stimulation in the home are related to positive academic achievement outcomes for African American adolescents. Punitive parenting appears to be unrelated to academic achievement. A more effective strategy appears to be cognitive stimulation at home, directly fostering learning through enriching materials and experiences, which was found to predict positive changes in academic performance across a two-year span.

This study possessed several strengths. It examined three types of parental involvement in education in African American families: punitive responses to lower-than-expected grades, nonpunitive responses to lower-than-expected grades, and cognitive stimulation in the home. Comparing the links between these types of parenting behaviors and adolescent achievement provides greater clarity regarding the types of parental involvement that are most helpful for student academic achievement. In addition, the longitudinal nature of this study allowed for an examination of the longer-term impact of parenting behaviors around school achievement. Further, this study adds to the literature on gender differences in parenting received by African American adolescents, pointing to another way in which boys and girls may experience differential socialization. Finally, beyond directions for future research, studies like this that isolate specific parenting behaviors can lead to more specific recommendations for academic counselors and policymakers regarding the relative benefit of cognitive stimulation compared to parental emotional reactions and punishments for lower-than-expected grades.

Despite the strengths of this study, results should be interpreted in light of some limitations. One limitation is that the items that comprised the measure of punitive parenting used in this study do not necessarily reflect severe or harsh punishments. Instead, this measure of punitive responses could reflect a broad range of behaviors, with some responses being relatively mild and other responses being more severe or harsh. As a result, the current study may have underestimated the association between punitive responses and academic achievement. A second limitation is that parents were asked to reflect on how they would respond to a hypothetical situation that may or may not have occurred in the past. Although parents’ reports likely indicate responses that are in their parenting repertoire, it is possible that parents may respond differently than what they indicated to actual incidences of low school performance. A third limitation is that self-report studies generally show fewer differences in how African American mothers parent boys and girls. Some differences in parenting related to child gender may be more readily detected with systematic observation than with self-report questionnaires (Mandara and Pikes 2008; Mandara et al. 2010). Additionally, although a standardized and well-known measure of cognitive stimulation was used in the study, an instrument that is more culturally relevant to African American families may have been even more sensitive to differences among families. Finally, the current study focused on low-income women who were adolescent mothers, which may raise concerns about the extent to which these findings are generalizable to other groups of mothers. Some work suggests that outcomes for adolescent mothers and their children are comparable to those of other women from similar SES backgrounds who delayed childbearing (SmithBattle 2009), supporting the generalizability of the findings to other low-income families.

The results of this study point to several directions for future research. There is a need for further study of differences in parenting based on child gender and how these differences may be linked to gender-based gaps in academic achievement. Future research should employ within-family designs that compare parenting of boys and girls within the same family, as within-family designs are better suited for detecting these types of effects than are between-family designs that compare parenting of boys and girls across families (McLoyd et al. 2019; Stanik et al. 2013). Research examining how parents actually respond to their children’s grades (rather than hypothetical grades) and whether these responses are related to academic achievement is warranted. Future studies that include more socioeconomically diverse African American parents and children are also needed to determine whether these findings hold across social class, as disciplinary strategies and beliefs about discipline vary by socioeconomic status (Greene and Garner 2012; Robinson and Harris 2013).

## Conclusion

This study investigated whether three forms of parental involvement, including cognitive stimulation in the home and punitive and nonpunitive responses to lower-than-expected grades, were related to low-income African American adolescents’ academic achievement. It further investigated gender differences in parental involvement and its relation to academic achievement. The results showed that nonpunitive responses to inadequate academic achievement were positively related to later academic achievement. However, this relationship became nonsignificant when prior levels of academic achievement (age 14) were controlled. Endorsement of punitive responses to inadequate academic achievement was not related to later academic achievement. Cognitive stimulation in the home was related to changes in academic achievement from 14 to 16 years of age, controlling for age 14 academic achievement. Finally, mothers of girls endorsed more punitive responses to grades than mothers of boys, but child gender did not moderate associations between parental involvement and academic achievement. The results suggest that cognitive stimulation in the home may matter more for promoting academic achievement than how African American parents respond to inadequate achievement.

## Footnotes

1 Previous versions of these analyses used a composite measure of academic achievement instead of a latent construct. The same structural model was tested, and the main results were similar. However, analyses with the composite measure had zero degrees of freedom, meaning that fit indices were not interpretable and chi-square difference tests could not be conducted.

2 In previous versions of these analyses, multiple group analysis to test for group differences in model parameters was conducted by imposing equality constraints on parameters across groups and using the chi-square difference test to compare models that have fewer equality constraints to models with more equality constraints. The analyses presented here directly test for significant differences in intercepts, means, and paths by sex instead of comparing models. Both approaches provide similar results; however, the approach used in the current study is more easily implemented in Mplus and provides results that can be interpreted more readily.

## References

[CR1] Addo F, Sassler S, Williams K (2016). Reexamining the association of maternal age and marital status at first birth with youth educational attainment. Journal of Marriage and the Family.

[CR2] Affuso G, Bacchini D, Miranda MC (2017). The contribution of school-related parental monitoring, self-determination, and self-efficacy to academic achievement. The Journal of Educational Research.

[CR3] Ansari A, Gershoff E (2016). Parent involvement in head start and children’s development: indirect effects through parenting. Journal of Marriage and Family.

[CR4] Baker, C. E., & Brooks-Gunn, J. (2019). Early parenting and the intergenerational transmission of self-regulation and behavior problems in African American Head Start families. *Child Psychiatry and Human Development*. 10.1007/s10578-019-00921-5. 10.1007/s10578-019-00921-5 31420763

[CR5] Banerjee M, Harrell ZAT, Johnson EJ (2011). Racial/ethnic socialization and parental involvement in education as predictors of cognitive ability and achievement in African American children. Journal of Youth & Adolescence.

[CR6] Benner A, Boyle A, Sadler S (2016). Parental involvement and adolescents’ educational success: The roles of prior achievement and socioeconomic status. Journal of Youth & Adolescence.

[CR8] Bradley RH, Caldwell BM (1984). The HOME Inventory and family demographics. Developmental Psychology.

[CR9] Bradley R, Corwyn R, Burchinal M, McAdoo H, Coll C (2001). The home environments of children in the United States Part II: relations with behavioral development through age thirteen. Child Development.

[CR10] Brody GH, Ge X, Kim SY, Murry VM, Simons RL, Gibbons FX, Gerrard M, Conger RD (2003). Neighborhood disadvantage moderates associations of parenting and older sibling problem attitudes and behavior with conduct disorders in African American children. Journal of Consulting and Clinical Psychology.

[CR11] Brown T, Linver M, Evans M, DeGennaro D (2009). African-American parents’ racial and ethnic socialization and adolescent academic grades: teasing out the role of gender. Journal of Youth and Adolescence: A Multidisciplinary Research Publication.

[CR12] Caughy MO, Mills B, Owen MT, Dyer N, Oshri A (2017). Ethnic differences in mothering qualities and relations to academic achievement. Journal of Family Psychology.

[CR13] Caughy MO, O’Campo P, Randolph S, Nickerson K (2002). The influence of racial socialization practices on the cognitive and behavioral competence of African American preschoolers. Child Development.

[CR14] Chapin LA, Altenhofen S (2010). Neurocognitive perspectives in language outcomes of Early Head Start: language and cognitive stimulation and maternal depression. Infant Mental Health Journal.

[CR15] Cho J, Kogan SM (2016). Risk and protetive processes predicting rural African American young men’s substance abuse. American Journal of Community Pyschology.

[CR16] Cornelius MD, Goldschmidt L, Day NL, Larkby C (2002). Alcohol, tobacco and marijuana use among pregnant teenagers: 6-year follow-up of offspring growth effects. Neurotoxicology and Teratology.

[CR17] Cornelius MD, Goldschmidt L, De Genna NM, Larkby C (2012). Long-term effects of prenatal cigarette smoke exposure on behavior dysregulation among 14-year-old offspring of teenage mothers. Maternal and Child Health.

[CR18] Dearing, E., & Tang, S. (2014). The promise of parent-school partnerships for narrowing the poverty achievement gap. In H. B. Weiss, M. E. Lopez, H. Kreider, & C. Chatman-Nelson (Eds). *Preparing educators to engage families: Case studies using an ecological systems framework* (3rd ed., pp. 100–104). Thousand Oaks, CA: Sage Publications.

[CR20] Dearing E, Wimer C, Simpkins SD, Lund T, Bouffard SM, Caronongan P, Weiss H (2009). Do neighborhood and home contexts help explain why low-income children miss opportunities to participate in activities outside of school?. Developmental Psychology.

[CR19] Deci EL, Ryan RM (2008). Facilitating optimal motivation and psychological well-being across life’s domains. Canadian Psychology/Psychologie canadienne.

[CR21] De Genna NM, Cornelius MD, Donovan JE (2009). Risk factors for young adult substance use among women who were teenage mothers. Addictive Behaviors.

[CR22] Duke DL (2017). Can within-race achievement comparisons help narrow between-race achievement gaps?. Journal of Education for Students Placed at Risk.

[CR23] Eamon MK (2005). Social-demographic, school, neighborhood, and parenting influences on the academic achievement of Latino young adolescents. Journal of Youth and Adolescence.

[CR24] Francesconi M (2008). Adult outcomes for children of teenage mothers. Journal of Economics.

[CR25] Greene K, Garner PW (2012). African American mothers’ disciplinary responses: associations with family background characteristics, maternal childrearing attitudes, and child manageability. Journal of Family and Economic Issues.

[CR26] Grolnick W, Raftery-Helmer J, Marbell K, Flamm E, Cardemil E, Sanchez M (2014). Parental provision of structure: implementation and correlates in three domains. Merrill-Palmer Quarterly: Journal of Developmental Psychology.

[CR27] Gutman LM, Peck SC, Malanchuk O, Sameroff AJ, Eccles JS (2017). Moving through adolescence: developmental trajectories of African American and European American youth: VI. Academic functioning. Monographs of the Society for Research in Child Development.

[CR28] Harris T, Sideris J, Serpell Z, Burchinal M, Pickett C (2014). Domain-specific cognitive stimulation and maternal sensitivity as predictors of early acdemic outcomes among low-income African American preschoolers. Journal of Negro Education.

[CR29] Hayes D (2011). Predicting parental home and school involvement in high school African American adolescents. The High School Journal.

[CR30] Hill NE, Castellino DR, Lansford JE, Nowlin P, Dodge KA, Bates JE, Pettit GS (2004). Parent academic involvement as related to school behavior, achievement, and aspirations: demographic variations across adolescence. Child Development.

[CR31] Hill NE, Tyson DF (2009). Parental involvement in middle school: a meta-analytic assessment of the strategies that promote achievement. Developmental Psychology.

[CR32] Jeon L, Buettner CK, Hur E (2014). Family and neighborhood disadvantage, home environment, and children’s school readiness. Journal of Family Psychology.

[CR33] Jeynes WH (2007). The relationship between parental involvement and urban secondary school student academic achievement. Urban Education.

[CR34] Jeynes WH (2016). A meta-analysis: the relationship between parental involvement and African American school outcomes. Journal of Black Studies.

[CR35] Joussemet M, Landry R, Koestner R (2008). A self-determination theory perspective on parenting. Canadian Psychology/Psychologie canadienne.

[CR36] Kazak AE, Brody GH, Yu T, Beach SRH, Kogan SM, Windle M, Philibert RA (2014). Harsh parenting and adolescent health: a longitudinal analysis with genetic moderation. Health Psychology.

[CR37] Lansford JE (2010). The special problem of cultural differences in effects of corporal punishment. Law and Contemporary Problems.

[CR38] Longo F, McPherran Lombardi C, Dearing E (2017). Family investments in low-income children’s achievement and socioemotional functioning. Developmental Psychology.

[CR39] Mandara J, Murray CB, Telesford JM, Varner FA, Richman SB (2012). Observed gender differences in African American mother‐child relationships and child behavior. Family Relations: An Interdisciplinary Journal of Applied Family Studies.

[CR40] Mandara J, Pikes CL (2008). Guilt trips and love withdrawal: does mothers’ use of psychological control predict depressive symptoms among African American Adolescents?. Family Relations.

[CR41] Mandara J, Varner F, Richman S (2010). Do African American mothers really ‘love’ their sons and ‘raise’ their daughters?. Journal of Family Psychology.

[CR42] McElhaney, K., Allen, J., Stephenson, C., & Hare, A. (2009). Attachment and autonomy during adolescence. In R. M. Lerner & L. Steinberg (Eds), *Handbook of adolescent psychology: Individual bases of adolescent development* (3rd ed., Vol. 1, pp. 358–403). Hoboken, NJ: Wiley.

[CR43] McKown C, Weinstein R (2008). Teacher expectations, classroom context, and the achievement gap. Journal of School Psychology.

[CR44] McLoyd, V. C, Hardaway, C., & Jocson, R. (2019). African American parenting. In M. H. Bornstein (Ed.), *Handbook of parenting: social conditions and applied parenting.* 3rd edn. (Vol. 4, pp. 55–107). New York, NY: Taylor & Francis/Psychology Press.

[CR45] Mueller, R. O., & Hancock, G. R. (2019). Structural equation modeling. In G. R. Hancock, L. M. Stapeton, & R. O. Mueller (Eds), *The reviewer’s guide to quantitative methods in the social sciences*. 2nd edn. (pp. 445–456). New York, NY: Routledge.

[CR46] Muthén, L. K., & Muthén, B. O. (1998–2017). *Mplus user’s guide.* 8th edn. Los Angeles, CA: Muthén & Muthén.

[CR48] Okano L, Jeon L, Crandall A, Powell T, Riley A (2020). The cascading effects of externalizing behaviors and academic achievement across developmental transitions: implications for prevention and intervention. Prevention Science.

[CR49] Pearman F, Curran F, Fisher B, Gardella J (2019). Are achievement gaps related to discipline gaps? Evidence from national data. AERA Open.

[CR50] Pomerantz EM, Moorman EA, Litwack SD (2007). The how, whom, and why of parents’ involvement in children’s academic lives: More is not always better. Review of Educational Research.

[CR51] Powell DR, Son S-H, File N, Froiland JM (2012). Changes in parental involvement across the transition from public school prekindergarten to first grade and children’s academic outcomes. The Elementary School Journal.

[CR52] Reardon SF (2016). School segregation and racial academic achievement gaps. The Russell Sage Foundation.

[CR53] Robinson K, Harris AL (2013). Racial and social class differences in how parents respond to inadequate achievement: consequences for children’s future achievement. Social Science Quarterly.

[CR54] Roche KM, Ensminger ME, Cherlin AJ (2007). Variations in parenting and adolescent outcomes among African American and Latino families living in low-income, urban areas. Journal of Family Issues.

[CR55] Roche KM, Ghazarian SR, Little TD, Leventhal T (2011). Understanding links between punitive parenting and adolescent adjustment: the relevance of context and reciprocal associations. Journal of Research on Adolescence.

[CR56] Royce EC (2015). Poverty and power: the problem of structural inequality.

[CR57] Schafer JL, Graham J (2002). Missing data: our view of the state of the art. Psychological Methods.

[CR58] Shanahan L, McHale SM, Crouter AC, Osgood DW (2007). Warmth with mothers and fathers from middle childhood to late adolescence: within- and between-families comparisons. Developmental Psychology.

[CR59] Shaw M, Lawlor DA, Najman JM (2006). Teenage children of teenage mothers: psychological, behavioural and health outcomes from an Australian prospective longitudinal study. Social Science & Medicine.

[CR60] Simpkins SD, Bouffard SM, Dearing E, Kreider H, Wimer C, Caronongan P, Weiss HB (2009). Adolescent adjustment and patterns of parents’ behaviors in early and middle adolescence. Journal of Research on Adolescence.

[CR62] SmithBattle L (2007). Legacies of advantage and disadvantage: the case of teen mothers. Public Health Nursing.

[CR63] SmithBattle L (2009). Reframing the risks and losses of teen mothering. The American Journal of Maternal/Child Nursing.

[CR64] Spengler M, Damian RI, Roberts BW (2018). How you behave in school predicts life success above and beyond family background, broad traits, and cognitive ability. Journal of Personality and Social Psychology.

[CR65] Spera C (2006). Adolescents’ perceptions of parental goals, practices, and styles in relation to their motivation and achievement. The Journal of Early Adolescence.

[CR66] Stanik CE, Riina EM, McHale SM (2013). Parent–adolescent relationship qualities and adolescent adjustment in two-parent African American families. Family Relations.

[CR68] Suizzo M, Jackson KM, Pahike E, McClain S, Marroquin Y, Blondeau LA, Hong K (2016). Parents’ school satisfaction and academic socialization predict adolescents’ autonomous motivation: a mixed-method study of low-income ethnic minority families. Journal of Adolescent Research.

[CR67] Suizzo M, Stapleton LM (2007). Home-based parental involvement in young children’s education: examining the effects of maternal education across U.S. ethnic groups. Educational Psychology.

[CR69] Tang S, Davis-Kean PE (2015). The association of punitive parenting practices and adolescent achievement. Journal of Family Psychology.

[CR70] Taylor, J., Kyere, E., & King, Ѐ. (2018). A gardening metaphor: a framework for closing racial achievement gaps in American public education system. *Urban Education*. 10.1177/0042085918770721.

[CR71] Tucker-Drob EM, Harden KP (2012). Early childhood cognitive development and parental cognitive stimulation: evidence for reciprocal gene–environment transactions. Developmental Science.

[CR72] Varner F, Mandara J (2013). Discrimination concerns and expectations as explanations for gendered socialization in African American families. Child Development.

[CR73] Varner F, Mandara J (2013). Differential parenting of African American adolescents as an explanation for gender disparities in achievement. Journal of Research on Adolescence.

[CR74] Vasquez A, Patall E, Fong C, Corrigan A, Pine L (2016). Parent autonomy support, academic achievement, and psychosocial functioning: a meta-analysis of research. Educational Psychology Review.

[CR75] Wang M, Hill NE, Hofkens T (2014). Parental involvement and African American and European American adolescents’ academic, behavioral, and emotional development in secondary school. Child Development.

[CR76] Wang M, Sheikh-Khalil S (2014). Does parental involvement matter for student achievement and mental health in high school?. Child Development.

